# 3D SHINKEI MR neurography in evaluation of traumatic brachial plexus

**DOI:** 10.1038/s41598-024-57022-0

**Published:** 2024-03-15

**Authors:** Yizhe Zhang, Xiaona Li, Ying Liu, Yingcai Sun, Luyao Duan, Yingshuai Zhang, Ruiqing Shi, Xiaoman Yu, Zhigang Peng

**Affiliations:** Department of Radiology, The Third Hospital of Hebei Medical University, 139 Ziqiang Road, Shijiazhuang, 050051 Hebei China

**Keywords:** Peripheral nervous system, Medical research

## Abstract

3D SHINKEI neurography is a new sequence for imaging the peripheral nerves. The study aims at assessing traumatic brachial plexus injury using this sequence. Fifty-eight patients with suspected trauma induced brachial plexus injury underwent MR neurography (MRN) imaging in 3D SHINKEI sequence at 3 T. Surgery and intraoperative somatosensory evoked potentials or clinical follow-up results were used as the reference standard. MRN, surgery and electromyography (EMG) findings were recorded at four levels of the brachial plexus-roots, trunks, cords and branches. Fifty-eight patients had pre- or postganglionic injury. The C5–C6 nerve postganglionic segment was the most common (average 42%) among the postganglionic injuries detected by 3D SHINKEI MRN. The diagnostic accuracy (83.75%) and the specificity (90.30%) of MRN higher than that of EMG (*p* < 0.001). There was no significant difference in the diagnostic sensitivity of MRN compared with EMG (*p* > 0.05). Eighteen patients with brachial plexus injury underwent surgical exploration after MRN examination and the correlation between MRN and surgery was 66.7%. Due to the high diagnostic accuracy and specificity, 3D SHINKEI MRN can comprehensively display the traumatic brachial plexus injury. This sequence has great potential in the accurate diagnosis of traumatic brachial plexus injury.

## Introduction

Trauma is a common clinical injury and the major cause of trauma in brachial plexus injury is mostly caused by car accidents, falls, sharp objects and machines. Conventionally, the diagnosis of brachial plexus injury mainly relies on clinical history, physical signs and electrophysiological examination^1^, but none of them can directly display the shape, injury site and extent of brachial plexus. MR has the advantages in high soft tissue resolution, multiplanar and no-invasion imaging. Studies have shown that it has become the preferred imaging modality for the diagnosis of brachial plexus injury^2,3^. MR neurography (MRN), as a specialized MR imaging technique for peripheral nerves, can well display the anatomical and pathological changes of peripheral nerves^4–8^, and is increasingly being used for routine clinical examinations^9,10^. Yoneyama et al. initially described the use of SHINKEI sequence in the brachial plexus and demonstrated its unique advantages in neural visualization^11^.

In this study, we retrospectively analyzed the MRN manifestations of patients with brachial plexus injury confirmed by surgery or clinical follow-up and aimed to explore the diagnostic value of 3D SHINKEI MRN for traumatic brachial plexus injury.

## Methods

### Participants

The study was approved by the Institutional Ethical Committee approval. Informed written consents from all the participants were obtained before the scans.

**Figure 1:**
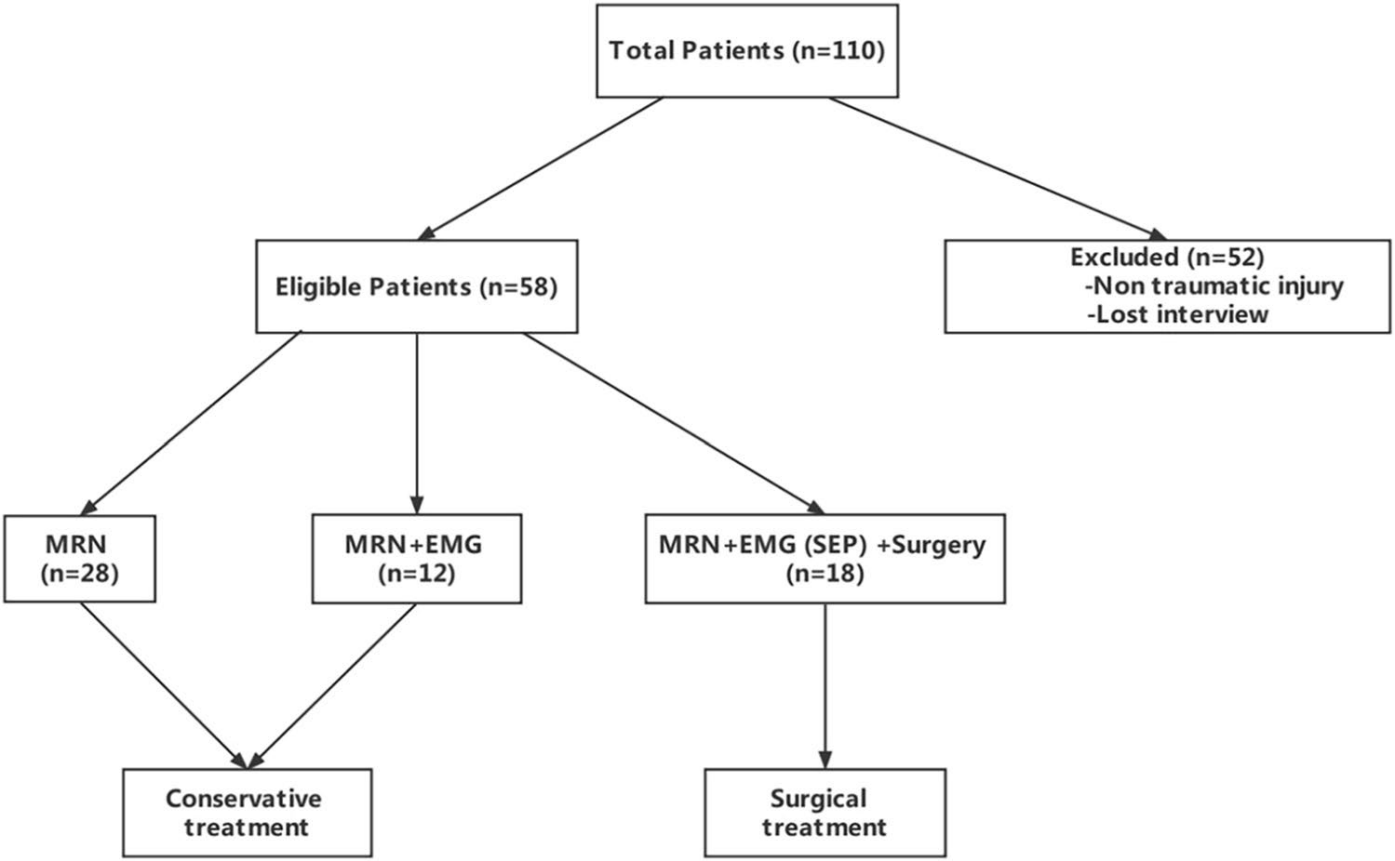
Flowchart shows selection and exclusion of study patients.

Patients were excluded if they could not complete the MRN examinations or MRN images could not fulfill the diagnostic criteria. These images usually have obvious artifacts, especially motion artifacts, or nerve injuries are masked due to swelling of surrounding soft tissue. The clinical data of 58 patients with brachial plexus injury who were treated for trauma from March 2021 to January 2022 were collected and compared. There were 48 males and 10 females with age rage 4–72 years and mean age of 40 ± 17. The causes of trauma were: 16 cases of traffic accident injury, 15 cases of fall injury, 5 cases of sharp objects injury, 2 cases of falling from a height, 2 cases of heavy object crushing injury, 2 cases of machine injury, 1 case of crush injury, 1 case of blow injury and 14 other cases with undefined cause of trauma. There were about 26 cases within 2 weeks, 12 cases in about 1 month, 17 cases in 2–6 months, and 3 cases in more than 6 months. All the 58 cases received MR scan. Of these, 30 cases underwent electromyography (EMG), 18 cases underwent surgical exploration or treatment, and 40 cases received conservative treatment, primarily consisting of physical therapy and neurotrophic medication (Fig. 1). The specific damage of the brachial plexus was assessed by the patient's clinical history (chief complaints, signs and symptoms), EMG (including intraoperative EMG), clinical follow-up (whether the condition improves after neurotrophic drug treatment), and (or) surgical confirmation.

Inclusion criteria:

1. Clinical diagnosis of brachial plexus injury is definitive.
2. All patients underwent MRN examination in our hospital.
3. Traumatic brachial plexus injury.

Exclusion criteria:

1. Non traumatic injury.
2. Unclear diagnosis.

Patients with suspected brachial plexus injury received MRN examination first. When MRN found suspected nerve rupture or patients with severe clinical symptoms (symptoms persist and difficult to alleviate), EMG examination was added; If both MRN and EMG strongly indicate nerve disruption (especially nerve preganglionic injury) or conservative treatment is ineffective, surgical exploration or repair should be taken. Three research groups (MRN, MRN + EMG, MRN + EMG + Surgery) were established according to whether the patients underwent MRN, EMG and surgery.

### MR imaging protocol and evaluation

MRN was performed on a 3.0 T MR system (Ingenia CX, Philips Healthcare, Netherlands). All the patients were kept in supine position with head advanced and arms by side, an eight-channel body coil was used, and rice bags were placed on both sides of the neck to decrease the susceptibility artifacts. Every patient was instructed to breathe gently and avoid swallowing and coughing as much as possible. The scanning area included neck and bilateral proximal humerus. MRN technology is consisted of 3D SHINKEI and T1-weighted (T1W) sequence in the coronal plane and 3D T2WI-driven equilibrium (DRIVE) TSE sequence in the axial plane (Table 1). 3D SHINKEI sequence was the study’s objective for evaluating the traumatic brachial plexus injury.

**Table 1:** Sequences and parameters for MR neurography.

Sequence	FOV (mm)	Slice thickness (mm)	Slice gap (mm)	TE/TR (ms)	Number of slices
3D SHINKEI	300 × 453	2.4	− 1.2	170/2200	80
T1WI	180 × 300	1.3	− 0.65	20/470	108
3D T2W DRIVE	150 × 150	3.0	− 1.5	100/1500	52

*DRIVE* driven equilibrium, *FOV* field of view, *TR* repetition time, *TE* time of echo, *T1W* T1-weighted, *T2W* T2-weighted.

After the raw data of 3D SHINKEI sequence was collected, multiplanar imaging reconstruction (MPR) and maximum intensity projection (MIP) (slice thickness of 20 mm, slice gap of − 18 mm and number of layers of 50) was used for image post-processing which could guarantee to observed the nerve shape, location, morphology and adjacency of brachial plexus in multiple angles and directions.

### Gold standard or the standard of reference

**Figure 2:**
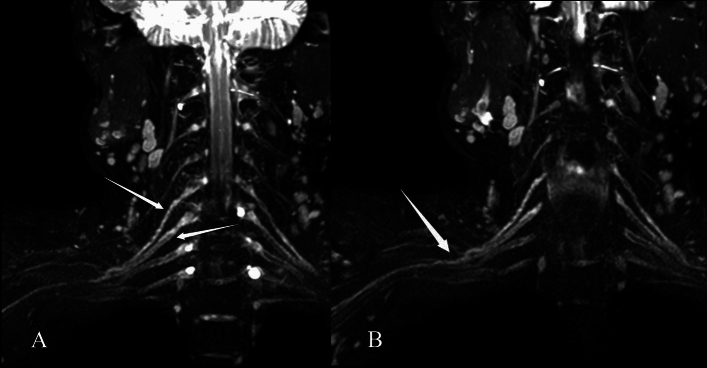
A 50-year-old man with right upper limb weakness for more than a month because of road traffic accident. Coronal 3D SHINKEI sequence shows (**A**) right postganglionic segment of C5–C6 and (**B**) upper trunk are slightly thicker and the signal is slightly higher (arrows). After surgical exploration, the patient found that the brachial plexus C5, C6, C7, upper trunk and middle trunk were compressed by scar tissue. MRN shows false negatives for some neural manifestations.

Surgical findings and intraoperative electrical stimulation results were used as the gold standard. All surgical patients underwent brachial plexus exploration. The suspected injured brachial plexus nerve was fully exposed during the operation. The surgeon was required to give feedback after visual inspection of the brachial plexus nerve. Any changes in the plexus’ typical appearance were regarded as a positive finding. For instance, scarring and/or fibrosis were described when the plexus lost its usual shiny aspect or when it felt hard to the touch^12^. This occasionally presents as a false negative on MRN (Fig. 2). In addition, the suspected injured nerve was detected by electrical stimulation, and the nerve functional integrity was judged by the contraction of innervated muscle.

**Figure 3:**
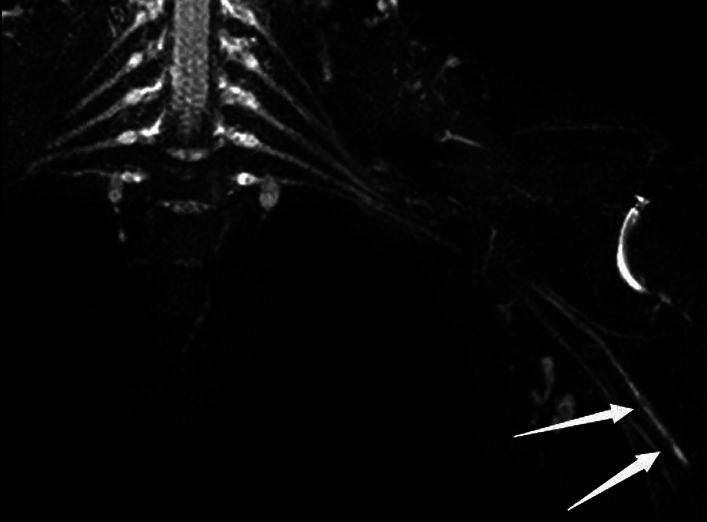
A 31-year-old man with history of left forearm scratch 1 month back. MIP reconstruction from coronal 3D SHINKEI sequence shows multiple hourglass—like changes of the left radial nerve (arrows). The patient underwent exploration and release of the radial nerve in the left upper arm. Intraoperative findings consistent with MRN.

Clinically relevant auxiliary examinations were taken as the reference standard for patients without surgery, mainly including MRN and EMG examination results. For MRN, the positive report would be given if there was a plexus rupture or a change in the morphology and/or signal of the plexus. The morphological anomaly showed that the nerve was thicker or thinned, while the signal abnormality showed that the T2 signal was raised or diminished, based on comparison with the patient's normal nerve on the opposite side. In addition, the comparatively infrequent nerve appearance was hourglass-like alterations (Fig. 3). In EMG, positive results were decreased or absent responses in innervation areas of the brachial plexus.

### Statistical analysis

The injured nerves detected by MRN were summarized and the frequency of the brachial plexus injury at each level was analyzed. The results of MRN and EMG were compared with that of surgery and intraoperative somatosensory evoked potentials or clinical follow-up results. The diagnostic accuracy, sensitivity and specificity were then calculated, respectively. The IBM SPSS Statistics version 26 (SPSS Chicago, USA) was used for all the statistical analyses and the count data was expressed by n (%). The *p* value < 0.05 was considered statistically significant. Observations within the scope of surgical exploration were used as the gold standard for comparison with MRN, and relative compliance with the MRN diagnosis was assessed on the basis of a rating system^12^. The rating was divided into A (complete agreement), B (basic agreement), C (partial agreement), D (no agreement), depending on whether the MRN findings are consistent with all four levels (root, trunk, cord, branch) or several levels related to surgical outcome (A for all, B for three or more than half of each level, C for at least one or less than half of each level, surgically detected and D as MRN not detected).

### Ethical board review statement

This material has not been published and is not under consideration elsewhere. This study is approved by the Medical Ethics Committee of the Third Hospital of Hebei Medical University and receives the financial support. All authors have confirmed that all methods were performed in accordance with the relevant guidelines and regulations. This study obtained the informed consent of all patients or their legal guardians, and all patients or their legal guardians agreed to participate in this study.

## Results

### Number of nerves detected by MRN

**Figure 4:**
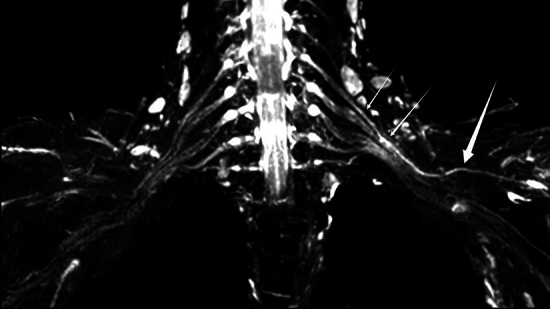
A 48-year-old man with history of road traffic accident 20 days back. Coronal 3D SHINKEI sequence shows T2 hyperintensity in left C5 posterior ganglion, upper trunk (small arrows) and suprascapular nerve (large arrow).

A total of 187 brachial plexus injuries were detected by MRN in 58 patients with brachial plexus injury. Among them, there were 15 roots in the preganglionic injury (4 roots were damaged at C5, 4 roots at C6, 3 roots at C7, 3 roots at C8, and 1 root at T1). The specific number of postganglionic injuries detected is shown in Table 2. No signs of injury were found in 6 cases. In addition, one case of unilateral suprascapular nerve injury was detected (Fig. 4).

**Table 2:** Imaging findings.

Injured nerve	Quantity		
	Left	Right	Total
C5	12	9	21
C6	11	11	22
C7	9	7	16
C8	5	7	12
T1	0	3	3
Upper trunk	8	8	16
Middle trunk	3	3	6
Lower trunk	0	4	4
Lateral cord	7	8	15
Posterior cord	1	5	6
Medial cord	0	3	3
Musculocutaneous nerve	5	6	11
Axillary nerve	6	9	15
Median nerve	2	4	6
Radial nerve	4	7	11
Ulnar nerve	1	4	5
Total	74	98	172

### MRN and EMG

**Figure 5:**
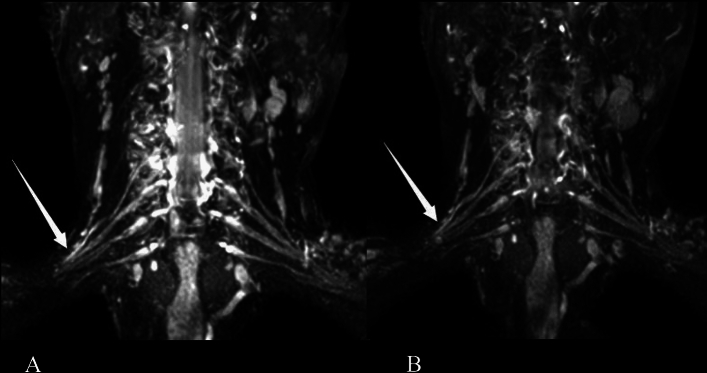
A 72-year-old man with history of a fall 4 months ago. Inadvertently discovered limitation of right upper arm abduction and lift 3 months ago. EMG shows damage to the upper trunk of the right brachial plexus. Coronal 3D SHINKEI sequence (**A**) and reconstructed MIP image (**B**) shows hyperintensity of the right C5 posterior ganglion and upper trunk without avulsion or discontinuity. The patient received non-surgical treatment and his condition slowly improved over the next few months. MRN provided reference for surgeons and altered the therapeutic decision to no surgery.

30 patients with brachial plexus injury underwent MRN and EMG examination. The correlation between MRN and EMG (Table 3) and their diagnostic results are shown (Tables 4 and 5). The diagnostic accuracy and specificity of MRN was higher than that of EMG (*p* < 0.001), and the sensitivity of EMG was higher than that of MRN (*p* > 0.05) (Fig. 5) (Table 6).

**Table 3:** Correlation between MRN and EMG.

Serial number	MRN	EMG	Clinical or SEP
1	Left C5–C7	Left total brachial plexus	Left C5–C6
2	Right C5–C6	Right total brachial plexus	RightC5–C7, upper/middle trunk
3	Left radial nerve	Left radial nerve	Left radial nerve
4	Left C5	Left five branches	Left C5–C6
5	Right upper trunk, radial nerve	Right radial nerve	Right radial nerve
6	Right radial nerve	Right C7–T1	Right radial nerve
7	Left C6–C7, upper/middle trunk	Left total brachial plexus	Left suprascapular nerve, upper trunk
8	Left C5–C8, upper/middle trunk, musculocutaneous/median/radial nerve	Left total brachial plexus	Left C5–C7
9	Left C5–C6, upper trunk, axillary/radial nerve	Left upper trunk, musculocutaneous/radial nerve	Left C5–C6
10	Right C5–C7, lateral cord, musculocutaneous/axillary nerve	Right total brachial plexus	Right C5–C7
11	Right C5, axillary nerve	Right total brachial plexus	Right C5–C6, upper trunk
12	Right upper trunk, axillary nerve	Right axillary	Right C5–C6, upper trunk
13	Right C5–C8, lateral/posterior cord, five branches	Right five branches	Right total brachial plexus nerves
14	Left C5–C8, lateral/posterior cord, C5–C6 (preganglionic)	Left total brachial plexus	Left C5–C7
15	Left C5	Left C5–C6	Left C5–C6
16	Left C5–C7	Left C5–C7	Left C5–C6
17	Right C5–C6, upper trunk, lateral cord	Right total brachial plexus	Right C5–C7, axillary/musculocutaneous nerve
18	Right C6–C7	Right radial/musculocutaneous/ulnar nerve	Right C5–C7, radial/musculocutaneous nerve
19	Left upper trunk, lateral cord, musculocutaneous/median nerve	Left musculocutaneous/median nerve	Left musculocutaneous/radial/median nerve
20	Normal	Left C7, median/ulnar nerve	Left ulnar/radial/median nerve
21	Normal	Right upper trunk, bilateral ulnar nerve	Right C5
22	Left C6, upper trunk, lateral cord, musculocutaneous nerve	Left axillary nerve	Left C5–C6, axillary nerve
23	Right C8, Lower trunk	Right C7–T1	Right C7–T1
24	Right total brachial plexus	Right total brachial plexus	Right total brachial plexus
25	Left C5, upper trunk	Left C5–C7, upper trunk	Left C5–C6, upper trunk
26	Right C5–C6, upper trunk, axillary nerve	Right C5–C6	Right C5–C6, upper trunk
27	Left C5–C6, lateral cord, musculocutaneous/axillary nerve	Left total brachial plexus	Left C5–C6, upper trunk, musculocutaneous nerve
28	Left C7, Middle Trunk, Lateral Cord, ulnar/musculocutaneous nerve	Left C7, Upper/Middle Trunk, ulnar/musculocutaneous nerve	Left C7, Middle Trunk, ulnar/musculocutaneous nerve
29	Left C5–C8 (preganglionic)	Left total brachial plexus	Left total brachial plexus
30	Normal	Normal	Rotator cuff injury

*MRN* magnetic resonance neurography, *EMG* electromyography, *SEP* somatosensory evoked potential.

**Table 4:** Diagnostic results of MRN (n = 480).

		Clinical positive (+)	Clinical negative (−)	Total
MRN	Positive (+)	76	35	111
MRN	Negative (−)	43	326	369
Total		119	361	480

*MRN* magnetic resonance neurography.

**Table 5:** Diagnostic results of EMG (n = 480).

		Clinical positive (+)	Clinical negative (−)	Total
EMG	Positive (+)	91	134	225
EMG	Negative (−)	28	227	255
Total		119	361	480

*EMG* electromyography.

**Table 6:** Diagnostic value of MRN and EMG n (%).

Inspection method	Accuracy	Sensitivity	Specificity
MRN	83.75 (402/480)	63.87 (76/119)	90.30 (326/361)
EMG	66.25 (318/480)	76.47 (91/119)	62.88 (227/361)
*X*^2^	54.301	3.050	19.547
*P*	< 0.001	0.081	< 0.001

*MRN* magnetic resonance neurography, *EMG* electromyography.

### MRN and surgery

**Figure 6:**
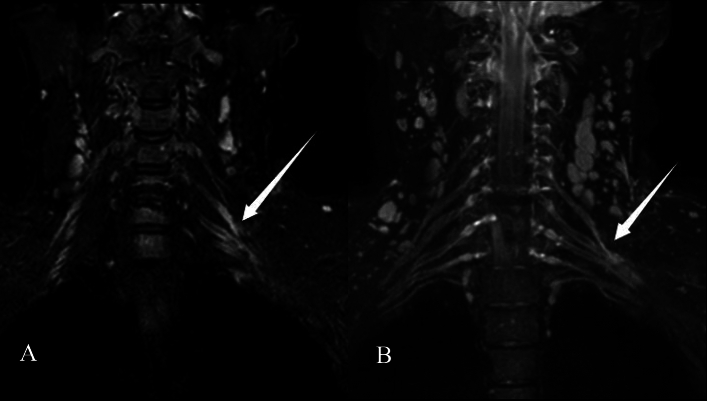
A 49-year-old woman with left-sided symptoms who underwent brachial plexus MRI following neck trauma. On the left C5 posterior ganglion and upper trunk, marked hyperintensity on coronal 3D SHINKEI sequence (**A**) and reconstructed MIP image (**B**) has been recorded (arrows). Surgery confirmed negative. This case has been considered a false positive.

Feedback was obtained from the operating surgeons, who were asked to record their findings at the root, trunk, cord, and branch levels (Table 7). The MRN examination's efficacy was graded using the rating system mentioned in the manuscript (Fig. 6) (Table 8).

**Table 7:** Compliance level of MRN to surgery.

Serial number	Surgical findings	MRN	Level
1	C5–C6	C5–C7	B
2	C5–C7, upper/middle trunk	C5–C6	C
3	Radial nerve	Radial nerve	A
4	C5–C6	C5	B
5	Radial nerve	Upper trunk, radial nerve	B
6	Radial nerve	Radial nerve	A
7	Upper trunk	C6–C7, upper/middle trunk	B
8	Left total brachial plexus	C5–C8, upper trunk, middle trunk, Musculocutaneous nerve, radial nerve, ulnar nerve	B
9	C5–C6	C5–C6, lateral cord, axillary nerve, radial nerve	C
10	C5–C6	Lateral cord, musculocutaneous nerve, axillary nerve	D
11	C5–C6, upper trunk	C5, axillary nerve	C
12	C5–C6, upper trunk	Upper trunk, axillary nerve	B
13	Lateral cord, posterior cord, five branches	C5–C8, lateral cord, posterior cord, five branches	B
14	C5–C7	C5–C8, lateral cord, posterior cord, C5–C6 (pre-segmental)	B
15	Lateral cord, median nerve, musculocutaneous nerve	C5–C8 and lateral cord	C
16	Normal	Lateral and posterior cords	D
17	C8 (avulsion), upper trunk	C8–T1 (pre-segmental)	B
18	Posterior cord, medial cord	Three cords	B

*MRN* magnetic resonance neurography.

**Table 8:** Efficacy of MR neurography as per operative findings.

A (completely consistent)	2 Patients
B (Basically consistent)	10 Patients
C (Partially consistent)	4 Patients
D (Non conformity)	2 Patients

## Discussion

The term “MRN” was first used in the early 1990s by Howe et al. to describe diffusion-weighted and fat-suppressed pulse sequences and applied to peripheral nerves^4^. MRN can directly show peripheral nerve abnormalities or local muscle denervation, therefore it is being increasingly used in clinical practice. In a recent study, Avneesh Chhabra et al. found that MRN could reduce unnecessary surgeries by 17%, and that MRN significantly influenced peripheral neurosurgeons' diagnostic thinking and treatment recommendations^13^. In recent years, MRN has been increasingly used to further evaluate suspected or confirmed brachial plexus and peripheral neuropathy with good results^14–17^. Abnormal MRN can be identified by differences in the course, thickness, contour of the brachial plexus and changes in signal intensity. Generally, the contralateral side is used as a reference for comparative observation. Although MRN has now developed into a highly sensitive technique, it still faces numerous diagnostic challenges. For example, when the brachial plexus signal increases slightly or is not obvious, it is difficult to define it as nerve damage; In addition, in some asymptomatic patients, mild intraneural T2 hyperintensity can sometimes be observed, which may represent subclinical neuropathy, and some studies have shown that it may be related to the magic angle effect^18^.

The 3D SHINKEI is a novel sequence that combines spectral adiabatic inversion recovery fat-compression and improved motion-sensitized driven equilibrium pre-pulse techniques^19^. By suppressing the surrounding background signals such as fat, blood vessels, lymph nodes, etc., this sequence can generate high quality neural images with higher resolution in 3.0 T high-field magnetic resonance imaging^16^, which can clearly display the postganglionic part of the brachial plexus (C5–T1, three trunks, three cords and five branches). After MRP, MIP and other post processing techniques, the course of the postganglionic brachial plexus can be clearly displayed from different angles, and abnormal lesions can be detected more sensitively by comparing the morphology and signals of the bilateral postganglionic brachial plexus. Direct signs of traumatic injury on 3D SHINKEI neurography images include the signal strength, nerve morphology, changes in continuity, the diameter and outer diameter are coarse or fine compared to the contralateral homonymous nerve in the patient. Indirect signs around the injured nerve including changes in the spinal cord (hemorrhage, edema, or displacement), soft tissue swelling, and increased signal intensity were observed. What's more, a clear history of trauma is useful in diagnosis. Especially, small nerves with the entire course and signal changes can be also better visualize, such as the suprascapular nerve which may be seen only in abnormal cases by 3D SHINKEI MRN^20^.

Nowadays, more and more patients have upper extremity sensory or motor impairment due to trauma such as car accident, and the limitation of upper extremity movement is common with brachial plexus involvement^21^. The brachial plexus is anatomically divided into five branches (C5–T1), three trunks (upper, middle and lower), six divisions (anterior and posterior), three cords (lateral, posterior and medial) and five branches (axillary nerve, musculocutaneous nerve, median nerve, ulnar nerve, radial nerve). The mechanism of traumatic brachial plexus injury in adults can be summarized into two types. One is that the high-speed injury makes the head violently deviate from the shoulder, which can lead to damage to the upper brachial plexus root, and the lower brachial plexus root will also be affected to varying degrees. The other is when the arm is violently abducted overhead, the injury may start in the lower part of the brachial plexus and then extend to the upper part^22^. In this retrospective study, most of the trauma causes were of car accidents and falls, which were more common in young and middle-aged men. Moreover most of them were postganglionic injuries of the brachial plexus. A study showed that the most common MRI feature of brachial plexus postganglionic injury is nerve trunk thickening and increased signal, accounting for approximately 41% of postganglionic injuries^23^. This was consistent in our study as well. All the trauma patients in our study underwent 3D SHINKEI MRN examination. By summarizing the number of injured nerves detected by MRN, we found that most of them were post-ganglionic injuries, a total of 172, accounting for 92%; pre-ganglionic injuries were 15, accounted for 8%. The C5–C6 postganglionic segment is the most common (average 42%) among postganglionic injuries, followed by the C7, upper trunk, lateral fascicle and axillary nerve (average 30%), the C8, radial nerve, musculocutaneous nerve (average 22%), and finally T1, middle trunk, inferior trunk, posterior cord, medial cord, median nerve, and ulnar nerve (average 6%). Thus, the easily damaged C5–C7, upper trunk, lateral cord, and axillary nerve are all worthy of attention in the future when using MRN in the diagnosis of traumatic brachial plexus injury. It would make brachial plexus diagnosis more purposeful and help to improve diagnosis accuracy by conduct screening and diagnosis based on the degree of vulnerability of each nerve. Despite C5–C6 nerve root avulsion is the most common preganglionic injury, followed by C7–T1, it is not discussed here due to the small sample size of preganglionic injury.

Electromyography is a supplementary approach used to diagnose brachial plexus injury. EMG can determine the degree of muscle denervation and quantify the number of functional nerves^24^. However, EMG may not reveal the morphological changes of injured nerves. In this retrospective study, 30 patients underwent EMG, and 119 nerve injuries were clinically confirmed, 89 nerve injuries were detected by MRN and 225 nerve injuries were detected by EMG. The diagnostic accuracy (83.75%) and the specificity (90.30%) of MRN higher than that of EMG. It can be seen that 11 cases of EMG were all diagnosed with brachial plexus injury. Combined with the clinical follow-up data and surgical records of the patients, at least 8 of them excluded the diagnosis of total brachial plexus injury. Therefore, EMG examination may exaggerate the actual nerve damage of the patient to some extent. In addition, there is often a certain latency period after nerve injury, and EMG requires at least 2–3 weeks to wait. For patients with short onset time and subacute neuropathy, EMG often shows false positives. MRN is frequently more visual for nerve injury than EMG and can offer comprehensive details of the nerves and surrounding tissues. When the brachial plexus injury is mild and there is only transitory nerve conduction malfunction, the patient's function can gradually improve over time, and if it can fully recover, no surgical intervention is needed. For such kind of damage, there might be no noticeable changes on MRN or simply a slight increase in signal strength and nerve edema. It will prompt to wait for a spontaneous recovery despite a still silent EMG or the absence of clinical improvement in the first few months after injury. The findings of this study also revealed that the use of MRN in patients with traumatic brachial plexus injury had no effect on the specificity of diagnosis when compared to EMG. In fact, EMG is often unreliable and irrelevant to take a clinical decision. Therefore, for the initial diagnosis of nerve injury, MRN can be used as the preferred method of examination.

Among the 58 patients whose clinical data were collected and reviewed, 18 underwent surgical exploration. The surgical methods were brachial plexus exploration and repair or brachial plexus exploration and release. All patients underwent 3D SHINKEI MRN examination before surgery. The results showed that there were 12 cases with a correlation between MRN and surgery with a rating of A or B. The number of injured nerves observed during surgery may be understated when compared to the actual number due to the restrictions of the surgical incision, or the probing site. The patient's number of damaged nerves means that the correlation between MRN and the actual damaged nerve of the patient will be higher than previously mentioned. Compared with surgery, MRN can obtain more comprehensive information. The degree of signal change of nerves on the image can prompt the surgical operation site, so that more serious areas are prioritized for exploration and repair. On the other hand, although the brachial plexus is basically included in the MRN view, it is a challenge for radiologists to evaluate it due to its complex anatomical structure and interference from various artifacts. In imaging studies, various muscular, vascular, and bony structures can be used as markers to identify different branches of the brachial plexus^25^. However, some blood vessels appear as high-brightness signals on 3D SHINKEI MRN. They are sometimes misdiagnosed as nerve damage because they are accompanied by peripheral nerves. The diagnosis should be given special consideration. In addition, MRI revealed swelling and thickening of the nerves in several patients who did not match the surgical findings, and the results of surgical exploration were negative. It is thought that the nerve damage is mild, the vision is insufficiently sensitive, and the MRN can clearly show small lesions^26^. The MRN was normal in several other patients, but the surgical exploration was positive, which may be a false-negative on the MRN due to scar formation around the nerve injury in the subacute stage after trauma.

### Limitations

The study had several limitations as follows. First, the sample size was limited, and the longest period of clinical follow-up did not exceed a year. Second, our study did not further categorize the types of surgery, for example, microsurgical dorsal root entry zone (DREZ) and the surgical control of suprascapular and axillary nerves. Third, as the MRN examination is relatively long, some patients are unable to cooperate well due to pain, resulting in the interference of factors such as motion artifacts, which affects the final diagnosis. Fourth, 3D SHINKEI MRN was ineffective in reducing fat in the lung's apical region, and some images did not clearly show the thoracic 1 nerve root.

## Conclusion

3D SHINKEI MRN can accurately and comprehensively display the brachial plexus, locate and describe the lesions from the nerve root to the five branches, thus providing a reliable imaging basis for clinical practice, which is conducive to the early diagnosis and treatment of patients with brachial plexus injury. It is an ideal imaging method that diagnosis to be wildly promoted and implemented in clinical practice for the diagnosis of traumatic brachial plexus injury.

## Data Availability

Data generated or analyzed during the study are available from the corresponding author by request.

## Abbreviations

3D SHINKEI: Three-dimensional nerve-SHeath signal increased with INKed rest-tissue RARE imaging
MRN: Magnetic resonance neurography
EMG: Electromyography
